# Revisiting the Timeline of Delayed Cerebral Ischemia After Aneurysmal Subarachnoid Hemorrhage: Toward a Temporal Risk Profile

**DOI:** 10.1007/s12028-022-01545-9

**Published:** 2022-07-06

**Authors:** Tobias Philip Schmidt, Miriam Weiss, Anke Hoellig, Omid Nikoubashman, Henna Schulze-Steinen, Walid Albanna, Hans Clusmann, Gerrit Alexander Schubert, Michael Veldeman

**Affiliations:** 1 Department of Neurosurgery, RWTH Aachen University Hospital, Aachen, Germany; 2 Department of Neurosurgery, Kantonsspital Aarau, Aarau, Switzerland; 3 Department of Diagnostic and Interventional Neuroradiology, RWTH Aachen University, Aachen, Germany; 4 Department of Intensive Care Medicine, RWTH Aachen University, Aachen, Germany

**Keywords:** Aneurysmal subarachnoid hemorrhage, Delayed cerebral ischemia, Timing, Outcome, Extended Glasgow Outcome Scale

## Abstract

**Background:**

Delayed cerebral ischemia (DCI) is one of the main determinants of clinical outcome after aneurysmal subarachnoid hemorrhage (SAH). The classical description of risk for DCI over time is currently based on the outdated concept of angiographic vasospasm. The goal of this study was to assess the temporal risk profile of DCI, defined by extended clinical and radiological criteria, as well as the impact the time point of DCI onset has on clinical outcome.

**Methods:**

All patients with aneurysmal SAH referred to a single tertiary care center between 2010 and 2018 were considered for inclusion. This study was designed as a retrospective cohort analysis and data were extracted from existing patient files. In conscious patients, DCI was diagnosed clinically, and in unconscious patients, diagnosis was based on perfusion computed tomography imaging and multimodal neuromonitoring. Extended Glasgow Outcome Scale scores were assessed after 12 months and compared between patients with early (< day 7) and late (≥ day 7) DCI onset.

**Results:**

The median delay from day of the hemorrhage (day 0) until detection of the first DCI event was 7.0 days, with an interquartile range of 5 days. The probability of DCI development over time demonstrated a bimodal distribution with a peak risk on day 5 (0.084; confidence interval 0.05.5–0.122) and a second peak on day 9 (0.077; confidence interval 0.045–0.120). A total of 27 patients (15.6%) suffered dominant hemispheric or severe bilateral DCI-related infarctions, resulting in the withdrawal of technical life support. Of those, the majority (20 patients, 22.2%) presented with early DCI onset (vs. late onset: 7 patients, 8.4%; *p* = 0.013).

**Conclusions:**

The risk profile of DCI over time mirrors the description of angiographic vasospasm; however, it comes with an added timely delay of 1 to 2 days. Early occurrence of DCI (before day 7) is associated with a higher infarct load and DCI-related mortality. Although the exact causal relationship remains to be determined, the time point of DCI onset may serve as an independent prognostic criterion in decision-making.

## Introduction

Aneurysmal subarachnoid hemorrhage (SAH) is a severe type of hemorrhagic stroke involving relatively young patients. The rupture of an intracranial aneurysmal causes blood to enter the subarachnoid space under arterial pressure, leading to a sudden increase of intracranial pressure. The resulting transient drop in cerebral blood flow sets off an elusive cascade leading to endothelial cell damage, microglial activation, and edema formation. These pathophysiological processes beginning 24 to 48 h after aneurysm rupture are coined as early brain injury and can contribute to later morbidity and mortality [1]. Delayed cerebral ischemia (DCI) has been identified as one of the main contributors to poor clinical outcome after SAH because it may, if left untreated, lead to cerebral infarction [2]. Initially believed to be the result of angiographic vasospasm, DCI is now perceived as a multifactorial concept [3]. Disruption of the blood–brain barrier, microthrombosis, and loss of autoregulation have been identified as contributing factors [4]. Reactive side products of hemoglobin degradation can induce vasoconstriction in animal experiments; however, strategies to reduce cisternal blood load in patients have had limited success in improving outcome [5, 6]. The role blood degradation products play in inducing DCI in humans is being questioned. Apart from blood degradation, no other pathophysiological mechanism exists to explain the delayed nature of these insults.

The time frame during which angiographic narrowing of cerebral arteries occurs after SAH is well described. Angiographic vasospasm generally starts around day 3 to 4 after aneurysm rupture, peaks around day 7 to 10, and resolves by day 14 [7, 8]. However, the concept of angiographic vasospasm leading to cerebral infarction in a delayed fashion is now considered an oversimplification. Congruous to a better understanding and the conceptualization of DCI, diagnostic tools assessing functional changes within the brain, such as metabolism and perfusion, are more commonly used. Perfusion computed tomography (CT) imaging and multimodal monitoring have replaced angiography for DCI detection in unconscious patients. Therefore, we revisited the timeline of DCI onset and its daily probability after aneurysm rupture in a setting in which this functional definition of DCI is applied. Furthermore, we aimed at assessing whether the timing of DCI onset affects the severity of further disease progression and responsiveness to treatment. We hypothesize that earlier DCI onset might reflect more severe underlying pathophysiology and be more detrimental. Finally, we aimed to identify the rate of late (post day 14) DCI and associated factors when this functional definition is applied.

## Methods

### Participants and Study Design

This study was designed as an observational cohort analysis and entails a subgroup of data that were partially previously published [9, 10]. This study was registered (ClinicalTrials.gov identifier NCT02142166) and approved by the Ethics Committee of the Medical Faculty of RWTH Aachen University (EK 062/14). Patients 18 years of age or older with confirmed SAH (either in CT angiography or digital subtraction angiography) who were referred to a single university hospital between 2010 and 2018 were included. Data have been prospectively collected since 2014, and written informed consent was obtained for these patients. Data of patients before 2014 were retrospectively extracted from digital hospital records. Patients with a moribund initial presentation (dilated pupils, other signs of brain stem herniation, or severe dominant hemispheric damage) and anticipated early mortality, SAH due to mycotic or traumatic/pseudoaneurysms, and aneurysms associated with arteriovenous malformations were excluded.

### SAH Treatment

After diagnosis, aneurysms were secured by either surgical clipping or endovascular occlusion within 48 h. The occlusion modality was based on interdisciplinary consensus. Each patient was observed in our neurointensive care unit for at least 14 days. The diagnostic and therapeutic treatment algorithm at our institution has been previously published in detail and is summarized below [11, 12].

In conscious patients, DCI was diagnosed clinically on the basis of the definition by Vergouwen et al. [13]. In unconscious patients not otherwise assessable, brain tissue oxygen probes (Neurovent PTO; Raumedic, Helmbrechts, Germany) and cerebral microdialysis catheters (71 High Cut-Off Brain Microdialysis Catheter; M dialysis, Stockholm, Sweden) were implanted in the watershed frontal region, ipsilateral to the ruptured aneurysm or highest blood load. Perfusion CT in conjunction with deterioration on multimodal neuromonitoring was used to define DCI in this cohort.

Induced hypertension (systolic blood pressure ≥ 180 mm Hg) via intravenous norepinephrine infusion was initiated in case of DCI-typical perfusion CT deficits (territorial cerebral blood flow/mean transit time mismatch) on the basis of neuroradiological consensus and/or oxygenation crises (brain tissue oxygen tension < 10 mm Hg) [14] or metabolic derangements (lactate/pyruvate ratio ≥ 40) [9, 15]. Onset of DCI was defined by the abovementioned extended criteria; however, for practical purposes, especially in respect to retrospectively collected data, the day of initiation of first tier treatment (induced hypertension) and the day of DCI diagnosis were equated. For all included patients, the binary outcome of DCI occurrence along its postictal day of onset was retrospectively reevaluated for each individual patient by two independent assessors (MV, MW), and discrepancies were discussed until consensus was reached.

In case perfusion deficits persisted under hypertensive treatment, endovascular rescue treatment consisting of transluminal balloon angioplasty or intraarterial spasmolysis was considered. For continuous local intraarterial nimodipine application, microcatheters (Excelsior SL-10; Stryker Neurovascular, Fremont, CA) were advanced into the internal carotid artery or vertebral artery and patients received weight-adapted intravenous tirofiban (Aggrastat; Correvia, Heathrow, UK). Treatment of DCI was reevaluated daily and deescalated as soon as possible. Tapering of induced hypertension was guided by imaging and multimodal monitoring.

### Assessed Variables and Outcome Definition

The primary outcome was defined as the extended Glasgow Outcome Scale (GOSE) score 12 months after the initial hemorrhage and dichotomized as unfavorable (GOSE scores 1–4) and favorable (GOSE scores 5–8) outcome [16]. Most outcome data were collected prospectively during scheduled follow-ups, but missing information was either appended by analysis of medical records or a structured telephone interview by a blinded assessor [17]. In many cases included before 2014, the GOSE score at 12 months had to be reconstructed retrospectively by investigation of medical records or by contacting the patient, the next of kin, or the caregiver in a structured telephone interview by an assessor blinded for additional clinical information. Secondary outcome included the incidence of DCI -related infarction and DCI-related mortality. DCI-related mortality was defined as the withdrawal of technological life support due to severe DCI-induced dominant hemispheric or bilateral infarction.

### Statistical Methods

Data are presented as the mean and standard deviation for continuous normally distributed variables. Nonnormal data are summarized as mean and interquartile range (quartile 1 to quartile 3). Categorical variables are provided as frequencies and proportions. After normality testing via the Shapiro–Wilk test, appropriate statistical tests were selected. Nominal data were tested with the χ^2^ test; for normally distributed continuous data, the unpaired *t*-test was used, and for nonnormally distributed data, the Mann–Whitney *U*-test was used. The Clopper–Pearson interval was used to calculate binomial confidence intervals (CIs) around daily DCI probability. All statistical analyses were performed using IBM SPSS Statistics 25 (SPSS Inc., Chicago, IL). Statistical significance was defined as a two-sided *p* value > 0.05.

## Results

### Patient Population

**Fig. 1:**
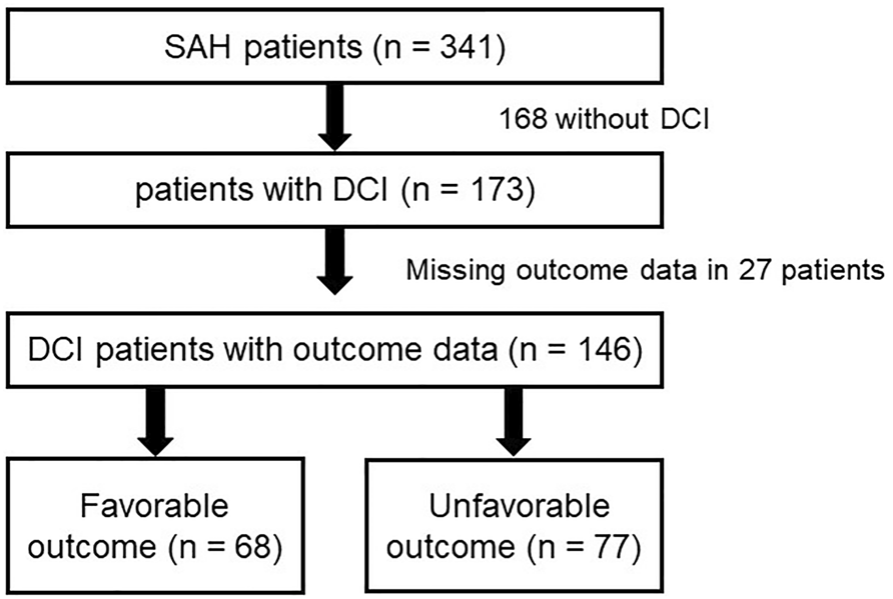
Inclusion flowchart. DCI delayed cerebral ischemia, SAH subarachnoid hemorrhage

The recruitment process is depicted in Fig. 1. A total of 341 patients with SAH were treated at our institution between 2010 and 2018. Twenty-four patients were excluded because of loss of follow-up. DCI was diagnosed in 173 (50.7%) patients, of whom 124 (71.7%) were women and 49 (28.3%) were men. The mean age of patients developing DCI was 54.0 ± 12.7. The GOSE score after 1 year was available in all but 26 patients. Relevant baseline characteristics are presented in Table 1.

**Table 1:** Comparison of patient- and SAH-specific characteristics between early (< day 7) and late (≥ day 7) DCI onset

	DCI (*n* = 173)	Early DCI (*n* = 90)	Late DCI (*n* = 83)	*p* value
Age (years), mean ± SD	54.0 (12.7)	52.5 ± 12.2	55.6 ± 13.1	0.106
Sex (female/male), no. (%)	124 (71.7)/49 (28.3)	65 (72.2)/25 (27.8)	59 (71.1)/24 (28.9)	0.868
Risk factors, no. (%)				
Hypertension	69 (39.9)	39 (43.3)	30 (36.1)	0.335
Smoking	59 (34.1)	22 (24.4)	37 (44.6)	**0.043**
Aneurysm location, no. (%)				0.235
Acomm	60 (34.7)	27 (30.0)	33 (39.8)	
MCA	49 (28.3)	24 (26.7)	25 (30.1)	
ICA	27 (15.6)	18 (20.0)	9 (10.8)	
Others	37 (21.4)	21 (23.3)	16 (19.3)	
Ant. circulation	148 (85.5)	78 (86.7)	70 (84.3)	0.663
Post. circulation	25 (14.5)	12 (13.3)	13 (15.7)	
Aneurysm size diameter maxium (mm), mean ± SD	7.1 ± 3.7	6.9 ± 3.5	7.2 ± 4.0	0.574
Hunt and Hess grade, no. (%)				0.301
Grade 1	19 (11.0)	11 (12.2)	8 (9.6)	
Grade 2	35 (20.2)	14 (15.6)	21 (25.3)	
Grade 3	56 (32.4)	33 (36.7)	23 (27.7)	
Grade 4	40 (23.1)	18 (20.0)	22 (26.5)	
Grade 5	23 (13.3)	14 (15.6)	9 (10.8)	
Intracerebral hemorrhage, no. (%)	77 (44.5)	39 (43.3)	38 (45.8)	0.746
Acute hydrocephalus, no. (%)	129 (74.6)	65 (72.2)	64 (77.1)	0.461
Modified Fisher scale, no. (%)				0.269
Grade 1	24 (13.9)	10 (11.1)	14 (16.9)	
Grade 2	17 (9.8)	12 (13.3)	5 (6.0)	
Grade 3	57 (32.9)	32 (35.6)	25 (30.1)	
Grade 4	74 (42.8)	36 (40.0)	39 (47.0)	
Aneurysm treatment, no. (%)				0.976
Clipping/endovascular	79 (45.7)/94 (54.3)	41 (45.6)/49 (54.4)	38 (45.8)/45 (54.2)	
DCI surveillance				
INM, no. (%)				0.735
ptiO_2_	73 (42.2)	39 (43.3)	34 (41.0)	
CMD	59 (34.1)	30 (33.3)	29 (34.9)	
Onset of first DCI event, days (IQR)	6 (4–9)	5 (3–5)	9 (8–10)	** < 0.001**

Bold values indicate *p* < 0.05

*Acomm* Anterior communication aneurysm, *CMD* Cerebral microdialysis, *DCI* Delayed cerebral ischemia, *ICA* Internal carotid artery, *INM* Invasive neuromonitoring, *IQR* Interquartile range, *MCA* Middle cerebral artery, *ptiO*_*2*_ Brain tissue oxygen tension, *SAH* Subarachnoid hemorrhage

### Risk of DCI Over Time

**Fig. 2:**
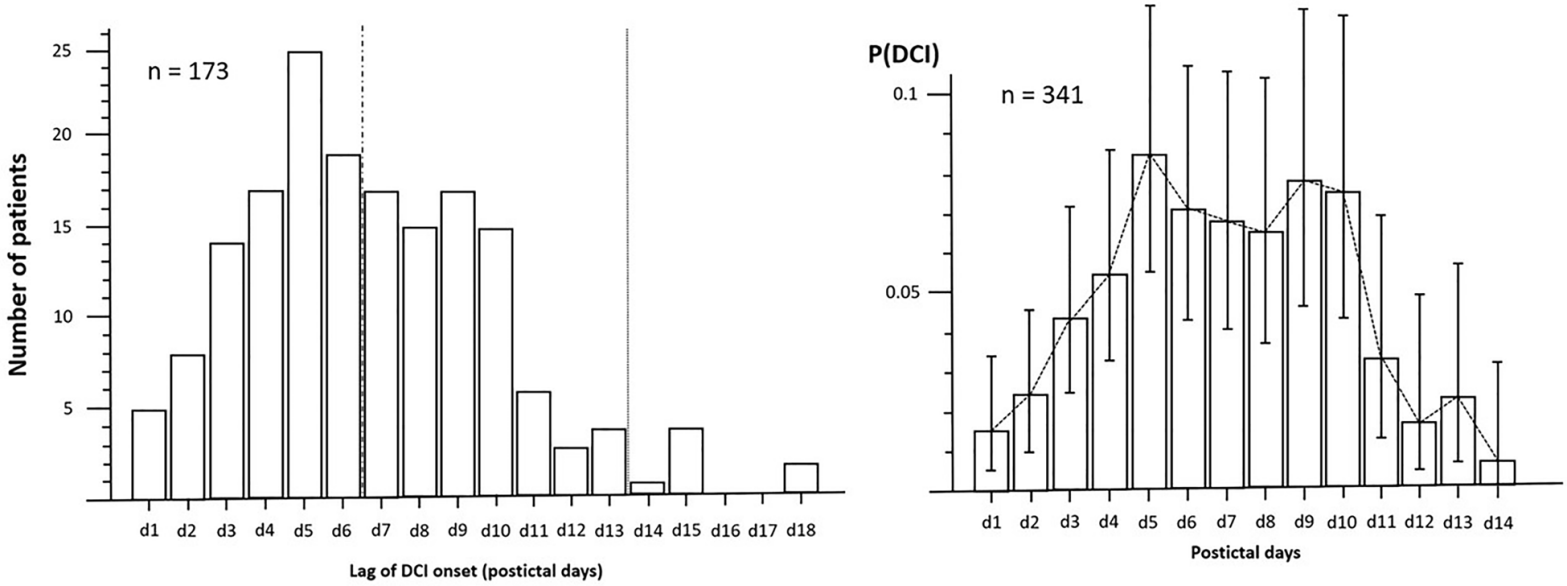
**a,** Prevalence distribution of DCI events over time in 173 patients. **b,** Probability distribution with confidence interval of DCI development per postictal day in 341 patients. DCI delayed cerebral ischemia, P(DCI), probability of DCI onset

The median time until detection of the first DCI event was 7.0 days (interquartile range 4–9). The plotted prevalence of DCI per postictal day is skewed to the right and drops considerably after day 10 in Fig. 2a. For each postictal day, the probability of DCI development, with 95% CIs, was calculated and is plotted in Fig. 2b. Risk of DCI over time demonstrated a bimodal distribution with a peak risk on day 5 of 8.4% (CI 5.5–12.2%) and a second peak on day 9 of 7.7% (CI 4.5–12.0%). The risk of DCI rapidly decreased after day 10 and bottomed on day 14 (0.6%; CI 0.0–3.1%).

### Early Versus Late DCI

The median (day 7) was used as a dichotomization point, dividing patients into early (< day 7) and late (≥ day 7) DCI onset. A total of 90 (52.0%) patients developed early DCI, whereas 83 (48.0%) patients were diagnosed with late DCI. A higher rate of smoking was observed in the late DCI onset subgroup (44.6% vs. 24.4%; *p* = 0.043). No other relevant differences in patient-specific and SAH-relevant baseline characteristics were identified between early and late DCI. Especially, SAH severity (clinically, Hunt and Hess and radiologically, modified Fisher) had no effect on whether DCI occurred before or after postictal day 7 (Table 1).

Early DCI onset was not associated with longer length of stay or higher rates of intensive care unit (ICU)-related complications. A total of 27 patients (15.6%) suffered dominant hemispheric or severe bilateral DCI-related infarctions, resulting in the withdrawal of technical life support. Of those, the majority (20 patients, 11.6%) presented with early DCI onset (late DCI onset: 7 patients, 4.0%; *p* = 0.013). Overall mortality figures, however, were not affected by the time point of DCI onset. Moreover, rates of favorable outcome after 12 months were comparable between early DCI onset and late DCI onset (41.1% vs. 37.3%; *p* = 0.987) (Table 2).

**Table 2:** Comparison of treatment characteristics and outcome between early (< day 7) and late (≥ day 7) DCI onset

	Early DCI (*n* = 90)	Late DCI (*n* = 83)	*p* value
DCI detection, no. (%)			
Clinical DCI	37 (41.1)	31 (37.3)	0.613
Metabolic DCI	39 (59.1)	35 (59.3)	0.979
Radiological DCI	71 (81.6)	73 (91.3)	0.071
Silent infarction, no. (%)	24 (26.7)	26 (31.3)	0.499
DCI treatment, no. (%)			
iHT	73 (81.1)	64 (77.1)	0.517
ERT	39 (43.3)	40 (48.2)	0.521
Spasmolysis	32 (35.6)	37 (44.6)	0.225
Continuous spasmolysis	17 (18.9)	12 (14.5)	0.361
Clinical outcome			
DCI-related infarction, no. (%)	46 (51.1)	43 (51.8)	0.972
Lag DCI-related infarction, mean ± SD	4.5 ± 1.7	9.6 ± 2.6	**< 0.001**
Favorable outcome (GOSE scores 5–8), no. (%)	37 (41.1)	31 (37.3)	0.987
Unfavorable outcome (GOSE scores 1–4), no. (%)	42 (46.7)	35 (42.2)	
Overall mortality, no. (%)	29 (32.2)	21 (25.3)	0.496
DCI-related mortality, no. (%)	20 (22.2)	7 (8.4)	**0.013**
ICU LOS, days ± SD	28.7 ± 17.5	30.9 ± 17.0	0.391
ICU-related complications, no. (%)			
Pneumonia	55 (61.1)	42 (50.6)	0.164
UTI	76 (84.4)	69 (83.1)	0.684
SIRS	53 (58.9)	42 (50.6)	0.274
Sepsis	24 (26.7)	15 (18.1)	0.177
Stunned myocardium	11 (12.2)	7 (8.4)	0.415
Cranial complications, no. (%)			
ICP crisis	33 (36.7)	27 (32.5)	0.517
DHC	24 (26.7)	22 (26.5)	0.981

Bold values indicate *p* < 0.05

*DCI* Delayed cerebral ischemia, *DHC* Decompressive hemicraniectomy, *ERT* Endovascular rescue treatment, *GOSE* Extended Glasgow Outcome Scale, *ICP* Intracranial pressure, *ICU* Intensive care unit, *iHT* Induced hypertension, *LOS* Length of stay, *SIRS* Systemic inflammatory response syndrome, *UTI* Urinary tract infection

### DCI Beyond Day 14

DCI was diagnosed in two (1.2%) patients after day 14. Clinically silent infarction was the first sign of ongoing DCI in four (2.3%) patients. Demographic data did not differ for patients with DCI or DCI-related infarction after day 14 compared with the remainder of patients. Also, no differences were identified regarding aneurysm location, size, occlusion modality, or hemorrhage severity in patients with DCI after day 14. Very late DCI onset did not contribute to a longer length of stay at the ICU (Table 3).

**Table 3:** Comparison of patient, SAH, and treatment characteristics alongside outcome between patients with very late DCI (> day 14) and the remainder of patients

	Ultralate DCI (*n* = 6)	Other patients with DCI (*n* = 167)	*p* value
Sex (female/male), no. (%)	4 (66.7)/2 (33.3)	120 (71.9)/47 (28.1)	0.782
Risk factors			
Age (years), mean ± SD	56.5 ± 19.2	53.9 ± 12.5	0.621
Hypertension, no. (%)	4 (66.7)	65 (38.9)	0.173
Smoking, no. (%)	2	57 (34.1)	0.968
Aneurysm location, no. (%)			
Acomm	3 (50.0)	57 (34.1)	0.296
MCA	3 (50.0)	46 (27.5)	
ICA	0 (0)	27 (16.2)	
Others	0 (0)	36 (21.6)	
Ant. circulation	6 (100.0)	142 (85.0)	0.306
Post. circulation	0 (0)	25 (15.0)	
Aneurysm size diameter maximum (mm), mean ± SD	8.2 ± 5.6	7.0 ± 3.7	0.471
Hunt and Hess grade, no. (%)			0.299
Grade 1	2 (33.3)	17 (10.2)	
Grade 2	0 (0)	35 (21.0)	
Grade 3	1 (16.7)	55 (32.9)	
Grade 4	2 (33.3)	38 (22.8)	
Grade 5	1 (16.7)	22 (13.2)	
Modified Fisher scale, no. (%)			0.571
Grade 1	2 (33.3)	22 (13.2)	
Grade 2	0 (0)	17 (10.2)	
Grade 3	1 (16.7)	56 (33.5)	
Grade 4	3 (50.0)	71 (42.5)	
DCI detection, no. (%)			
Clinical DCI	3 (50.0)	65 (38.9)	0.585
Technical DCI	3 (50.0)	102 (61.1)	
Silent infarction, no. (%)	4 (66.7)	46 (27.5)	**0.038**
DCI treatment, no. (%)			
iHT	4 (66.7)	133 (79.6)	0.442
ERT	2 (33.3)	77 (46.1)	0.537
Spasmolysis	2 (33.3)	67 (40.1)	0.709
Continuous spasmolysis	0 (0)	29 (17.4)	0.330
Clinical outcome			
DCI-related infarction, no. (%)	4 (66.7)	85 (50.9)	0.448
Lag DCI-related infarction, mean ± SD	15.3 ± 3.4	6.8 ± 2.9	**< 0.001**
Favorable outcome (GOSE scores 5–8), no. (%)	2 (33.3)	65 (38.9)	0.876
Unfavorable outcome (GOSE scores 1–4), no. (%)	2 (33.3)	74 (44.3)	
Overall mortality, no. (%)	1 (16.7)	49 (29.3)	0.354
DCI-related mortality, no. (%)	0 (0)	27 (16.2)	0.284
ICU LOS, days ± SD	41.7 ± 25.4	29.3 ± 16.9	0.085
Length of ventilation	23.8 ± 20.1	21.5 ± 16.2	0.289
ICU-related complications, no. (%)			
Pneumonia	3 (50.0)	94 (56.3)	0.760
UTI	6 (100.0)	139 (83.2)	0.282
SIRS	3 (50.0)	92 (55.1)	0.806
Sepsis	1 (16.7)	38 (22.8)	0.726
Stunned myocardium	1 (16.7)	17 (10.2)	0.609
SAH-specific complications, no. (%)			
Hydrocephalus	3 (50.0)	126 (75.4)	0.160
ICP crisis	0 (0)	60 (35.9)	0.184
DHC	0 (0)	46 (27.5)	0.132

Bold values indicate *p* < 0.05

Acomm, anterior communication aneurysm, *DCI* Delayed cerebral ischemia, *DHC* Decompressive hemicraniectomy, *ERT* Endovascular rescue treatment, *GOSE* Extended Glasgow Outcome Scale, *ICA* Internal carotid artery, *ICP* Intracranial pressure, *ICU* Intensive care unit, *iHT* Induced hypertension, *LOS* Length of stay, *MCA* Middle cerebral artery, *SAH* Subarachnoid hemorrhage, *SD* Standard deviation, *SIRS* Systemic inflammatory response syndrome, *UTI* Urinary tract infection

## Discussion

DCI has been thoroughly reconceptualized as a multifactorial process in which macrovasospasm is only a contributing factor. New insights in DCI pathophysiology have broadened diagnostic criteria and made way for the application of additional diagnostic tools, i.e., perfusion CT imaging (typically triggered by transcranial Doppler), cerebral microdialysis, or brain oxygenation monitoring [9, 13]. In contemporary large SAH series in which DCI is assessed beyond angiographic vasospasm, the timeline of its onset is not addressed [2, 18].

We applied a combined clinical and functional DCI definition that included perfusion CT imaging. The risk of DCI presented as a bimodal distribution, with the highest risk on day 5 and a second peak on day 9 to 10. This mirrors the description of angiographic vasospasm but with an additional delay of 1 to 2 days. Earlier DCI onset was associated with a higher rate of DCI-related death because of more pronounced infarct load. It appears treatment in patients with early DCI proved not as effective in the prevention of severe infarction. This confirms the notion that earlier DCI may be an outing of more sever underlying pathophysiology. Early DCI onset presents a negative prognostic marker independent of clinical or radiological hemorrhage severity. DCI beyond day 14 was extremely rare and was observed only in 3.5% of cases. Of those, two thirds presented with already demarcated infarction, suggesting that the process of DCI started earlier. We were not able to identify any patient- or SAH-specific factors associated with very late DCI onset. Diagnosis of DCI after 14 days contributed to neither a longer ICU length of stay nor worse clinical outcome.

The classical understanding of the delayed nature of angiographic vasospasm is based on blood degradation and the time it takes for oxygen radicals to build up in the subarachnoid space. Hemolysis of red blood cells starts 16 to 32 h after aneurysm rupture and peaks around day 7 [19]. Although the exact spasmogenic agent has not been identified yet, the vasoconstrictive effect of blood degradation products has been well documented in animal SAH models [5]. In humans, efforts to increase the rate of subarachnoid blood clearance have had limited effect on outcome [6, 20].

In its most simplified form, DCI represents (still reversible) decompensation in oxygen/glucose supply and demand. Contribution factors can either cause decreased supply or increased demand. Angiographic macroscopic vasospasm causing decreased supply can largely be compensated as substantiated by the high rate of vasospasm remaining asymptomatic. Much more difficult to contemplate are microvascular changes within the neurovascular unit. Constriction of the microvasculature is consistently observed in experimental SAH models and remains irresponsive to induced hypertension [21]. A potassium surge drives inverted neurovascular coupling, creating a vicious circle in a brain where inflammatory repair mechanisms have already increased energy demand [22]. Local hypercoagulability and endothelial changes allow for formation of microthrombi, further reducing supply, and their presence has demonstrated spatial correlation with regions of neurol apoptosis [21, 23].

Neuroinflammation and the time required for the recruitment of circulating white blood cells, as well as buildup of a microglial response, offers an alternative explanation toward the delayed nature of DCI [24]. Inflammatory reactive changes in the central nervous system after SAH are incompletely understood but can be indirectly observed by a systemic increase of acute phase reactants, such as C-reactive protein and procalcitonin [25, 26]. Additionally, cerebrospinal fluid concentrations of proinflammatory cytokines, such as interleukin-1β, interleukin-6, interleukin-8, and tumor necrosis factor α, correlate with DCI and poor outcome [27–29]. Although immunosuppressive treatment, such as glucocorticoid use, has had no effect on DCI and outcome so far [30].

A possible explanation for milder ischemic injury in late DCI onset might be the longer recovery time the brain has after early brain injury before a second hit. After the initial insult, activated inflammatory cells become readily susceptible and primed for any subsequent cue. Consecutive stimuli can have a synergistic and potentiating effect on neuroinflammation, increasing tissue damage [31, 32]. Such a response is observed in traumatic brain injury, in which an early second hit can cause disproportional amounts of additional damage [33–35]. We speculate that this double-hit hypothesis can be an explanation for the increased mortality rate after early DCI, although future studies are needed to verify this concept.

An extended definition of DCI encompassing these changes, as well as allowing evaluation of unconscious patients, is urgently needed. Our compound definition of DCI highly relies on perfusion CT imaging. A relevant limitation thereof is the existing broad heterogeneity in perfusion CT definitions of DCI along with ubiquitous differences in image acquisition protocols and postprocessing algorithms. The definition of DCI in perfusion CT requires standardization before inclusion of this diagnostic modality in a newly phrased extended DCI definition, which is application for unconscious patients. Moreover, the lack of incorporating change over time of brain oxygenation and microdialysis parameter should be addressed as a limitation.

Despite rigorous documentation, the retrospective nature of this study allows for information bias to be introduced. Attrition bias due to loss to follow-up is suspected to have limited effect on results because loss to follow-up was equally distributed between early and late DCI. The accuracy of time specification for day 0 (day of hemorrhage) can be biased by aneurysm rupture earlier than the day of admittance. Whenever possible, the day of ictus was reconstructed on the basis of the patient’s history. Our applied functional DCI definition is heterogeneous and not yet widely accepted and most probably accounts for the high rate of DCI in our cohort. Outcome data were partially retrospectively assessed, creating potential observer and information bias.

## Conclusions

The timeline of functional DCI mirrors that of angiographic vasospasm, with an additional delay of 1 to 2 days. Early onset of DCI is associated with a higher risk of severe cerebral infarction. Although the exact causal relationship remains to be determined, the time point of DCI onset may serve as an independent prognostic criterion in decision-making.

## Clinical trial registration

The data collection process was part of a previously registered observational study (ClinicalTrials.gov identifier NCT02142166).
